# Supplementary material for: Interactive effects of discharge reduction and fine sediments on stream biofilm metabolism
Source: PLoS One. 2021 Feb 11;16(2):e0246719. doi: 10.1371/journal.pone.0246719 (PMC7877786; doi:10.1371/journal.pone.0246719)
Supplement: Attachment — Submitted filename: Response to Reviewers.docx [file pone.0246719.s002.docx]

Dear Dr. McNair,

A revised version of the manuscript PONE-D-20-22278 “Interactive effects of flow reduction and fine sediments on stream biofilm metabolism”, now entitled “Interactive effects of discharge reduction and fine sediments on stream biofilm metabolism” by Ana Victoria Pérez-Calpe, Aitor Larrañaga, Daniel von Schiller and Arturo Elosegi, is attached to your consideration for publishing as an article in PLOS ONE journal.

We are very grateful to the reviewers for their detailed and positive comments. We have followed closely these comments, as we detail below.

We hope that the present version of the Ms reaches the standards for PLOS ONE journal.

Best regards,

Ana Victoria Pérez-Calpe, PhD student

Faculty of Science and Technology

University of the Basque Country,

P.O. Box 644, 48080 Bilbao, Spain

Tel.: +34 94 601 5939

Email: anavictoria.perez@ehu.eus

**Comments to the Author**

Reviewer #1: This paper contributes to our understanding of sediment impacts on stream ecosystems, an understanding that is surprisingly limited given the magnitude of the issue. It is a strong paper in many ways: there are clearly stated hypotheses, a carefully designed well-replicated split plot experiment, an impressive test facility, and effective measurement of relevant variables. I have concerns, however, about how the results are interpreted in the context of previous work and of the limitations of the study design. The study found that sediments increase benthic biomass and productivity, and that (in the absence of added sediments) there is negative relationship between benthic biomass and water velocity. These are potentially useful results, but both supporting and contradictory results appear in the literature. The authors do cite results from both sides, and they acknowledge that, “[t]he response of biofilm to flow reduction and fine sediment deposition can be complex” (line 67). But their effort to resolve these complexities is inadequate. The authors further found that, in the presence of added sediments, their observed negative effect of velocity disappeared. This is perhaps the only truly novel result of the study yet their attempt to explain it (lines 296-298) falls short. I return to these concerns with more specifics in the comments that follow.

The term “flow reduction” is confusing in that a positive response to flow reduction is a negative response to flow. Further, the experiment involved both increases and decreases relative to the acclimatized flow. I would limit the use of “flow reduction” to the introduction and discussion.

**Response: We followed the advice of the reviewer and now talk of "discharge" instead of "discharge reduction". The latter is only used in the Introduction, to frame the experiment in the context of alterations in discharge, especially reductions caused by water diversion.**

Line 35: Unclear. How about “ranges of … and of” rather than “ranges from … and from”

**R: Modified. Line: 37.**

Lines 69-72. Too many reversals: “Conversely…On the other hand…However”

**R: Modified.**

Line 78. Missing here is a transition from the general problem to the specific design. What answers were being sought that motivated this particular experiment (these particular hypotheses)?

**R: Added in the text. Line 78.**

Line 95: change to “filtered (1 mm) rainwater”

**R: Modified. Line 95.**

Line 95: How fast was the water fed from the primary tank to the block tanks? Or did the system run closed for extended periods, renewed in batch on days 7 and 14 (line 137)? I wanted to see this to infer the mass of sediment actually added and to interpret the water quality data vis a vis water renewal.

**R: Blocks ran as closed system for periods of 7 days, and for this reason, day 7 and 14 water was renewed. We have clarified this in the text. Line 97.**

Line 95: A diagram of the set-up (like Matthaei et al. 2010, Fig 1) would help a lot. I’ve had to assume that all recirculation passed through the block tank and that there were not separate overflows from each channel. I’ve just now seen that is likely the system ran closed.

**R: The reviewer is right that all channels in the same block received the water from the same tank. A diagram has been included as Fig 1. Line 97.**

Line 128: We need a conversion of the “lixiviates” (a new term for me) into mass of nutrients that were potentially available to the biofilms. E.g., the solid:liquid ratio represented by these concentrations, and then the mass of sediments transferred to the channels. This is necessary to evaluate the inference that the sediments stimulated the biofilm through nutrient release. More below.
**R: Sorry, it was a mistake. We have changed the term “lixiviate” to “leachate”. Moreover, we now provide the characterization of the leachates (mg/g of sediment) and the mass of nutrients they provided to each tank. We could not calculate the mass of nutrients transferred to each channel, since the fact that channels in the same block were all fed with water from the same tank determines that the dissolved nutrients were those leached from the total sediments in the block, not from the sediments retained in each channel. Line 135.**

Line 136: Apparent discrepancies: As the sentence reads, I count six, not four, sample times. There are five sets in the Supplementary Data. If the numbers under “Sampling” in the Supplement represent experiment days, these do not match the description on lines 136-137.

**R: Sorry, the reviewer is right, there were six samplings. We have corrected the text and the supplementary data and Table 1 has been updated.**

Line 171: The conversion of 0.0036 mg/(L-NTU) seems way too low. It usually runs between 1 and 2.
**R: Sorry, there was a mistake in sediment concentration units. It has been corrected. Line 178.**

Line 177: Insert abbreviation “(LMEM)”

**R: Done.**

Lines 187-203 (Water Quality). The potential for nutrients to have stimulated benthic biomass raises the question of whether there was a temporal signal in the water quality data. It appears to me from the Supplemental Data that there was not, but perhaps the timing of the WQ samples was such that a short-term pulse would have been seen. This issue should be addressed in the text, if only to say that none was detected and perhaps why none was detected. The Supplemental Data should clearly identify the sampling date.
The data for sediment deposited in the Supplemental Data are ten times higher than in Figure 1B. Were these in g/m2 but mislabeled as mg/cm2?

**R: Water renewal caused some small changes in water quality. It has been added in text. Line 204.**

**Sampling dates has been included in supplementary data and the mistake in the magnitude of sediment deposition has been corrected.**

Line 206: Strike “Results from the LMEM showed that”. LMEM is made clear in Table 2.

**R: Done.**

Line 207: Strike “LMEM determined that”

**R: Done.**

210: Did deposited sediment really increase with flow in the no-sediment treatment? The broken line in Fig. 1B is nearly horizontal. The R2=0.44 strikes me as impossible. I would guess 0.01.
**R: Thanks to the referee comment, we noticed that showing R2 and ANOVA information could be confusing, and we decided to remove the R2 and only use the information provided by ANOVAs. Thus, Fig 1A and 1B (now renamed Fig 2A and 2B) have been modified in this sense.**

**In Fig 1C (now Fig 2C) to avoid misunderstanding with solid and dotted lines of trend, we have eliminated them.**

211-213: Admitting I am not a statistician, I would say that the interaction simply verified that the flow effect on deposition differed by treatment.

**R: Modified. Line 320.**

Lines 214-221 (Table 2). Could be simplified. The F-value could be eliminated as (nearly) redundant on P-value. I see no value in the intercept statistics. Thanks for the d.f.s. They are important.
**R: We eliminated the intercept. On the other hand, we find the F-value is necessary as it is a standard way to compare the variability among treatments with the variability within. Therefore, we prefer to maintain it.**

Line 231: As noted above, it would help to frame responses in terms of flow increase, rather than decrease.

**R: Results section have been rewritten in terms of flow increase.**

Line 237: Replace “scouring” with something else. “negative”? I don’t think you were observing scouring; perhaps sloughing or detachment of strands.
**R: Sorry, our mistake. We have replaced the term “scour” with “slough” in all the text.**

Line 243: Replace “rate” with “metric”, “parameter”, or something. “rate” is confusing here because it suggests that rates were quantities.
**R: We have replaced “metabolic rate” with “metabolism metric” in all the text.**

Lines 244-245: The regression lines in Figure 3 do not fit the data. I regressed the (log) relationships from the Supplemental Data. The no-sediment line has a significant (P~0.025) upward slope and an R2 of 0.35. The sediment line has a near-zero slope and an R2 of 0.01. This affects interpretation. Flow has no effect in the presence of sediments. Metabolic efficiency increases with flow in the absence of sediments. At low flows, metabolic efficiency is higher in the presence of sediments than in their absence, but the two are equal at high flows. Basically what we see here is (1) the inverse of the no-sediment chlorophyll-flow relationship (i.e., since chlorophyll declined but GPP didn’t, metabolic efficiency increased), together with (2) an enhancement of metabolic efficiency at all flows in the presence of sediment (GPP increased more than chlorophyll).

**R: Thanks to the reviewer comment, we noticed that the dotted slope line was incorrect. We have modified the Fig 3 (now renamed Fig 4). Moreover, interpretation has been changed in the discussion section. Line 325.**

Lines 251,252: Strike “When” and end the sentence at “significant”.

**R: Done.**

Lines 259,260. I suggest: “biomass, and their interaction was not additive but antagonistic.” “Antagonistic” is a technical term in statistics but not all readers know this.

**R: Text has been rewritten avoiding “antagonistic” and “synergistic” terms to improve understanding.**

Line 255 (DISCUSSION). You need to address why sediment deposition increased with water flow. This result is presented in the abstract and shown prominently as Fig. 1 B. I was at first puzzled, thinking that more should deposit at the slower velocity, until I realized that the greater delivery at higher flows would have overridden this effect. The tight correlation between deposited sediment and water velocity is potentially important: it means that any response to water flow observed in sediment-treated mesocosms would have been indistinguishable from a response to the quantity of deposited sediment. Fortunately, there were no such responses.

**R: Done. Lines 126 and 226.**

Lines 265-267 (Discussion of velocity effects). First, some corrections. On line 270, “above” should read “to” or better yet, delete after “declined” because this referred to a different life form, one that had no optimum. Use consistent units, which here would be cm/s. Thus, Biggs’ optimum was 20 cm/s. Honzo and Wang’s optimum was 15 cm/s, not 0.7 cm/s which is a shear velocity not a water velocity.

I think this section should be rewritten to clarify. Point out that many studies have observed an optimum velocity, that these are typically in the range of 20 cm/s or more, but that your results suggest either an extremely low optimum (< 3 cm/s) or none at all, which is consistent with Lau and Liu, and with the result of Biggs et al. for long filamentous forms. You then need to speculate on why your result is unusual – were there long filamentous forms? You should not dismiss your result (“we did not observe any major effect”) by appealing to the small velocity range, because it is a central result of the study.
**R: Done. We have rewritten the section following the reviewer suggestions and we have focused the discussion on biofilm growth form. Lines 280-296.**

Lines 277-288 (Discussion of sediment effects). The unexpected result that sediments enhanced biofilm growth should be treated with care because it could be used to downplay the need to control sediment inputs. The claim that the result “is line with findings of other mesocosm studies” should be modified to acknowledge that it is not in line with most mesocosm studies. Of the two cited, Izagirre found that growth rates were below control for higher additions. I agree that for your case, it is probably nutrients that explain the increase, but the citation to Magbanua is wrong: they ruled out nutrients and went with Izagirre’s habitat argument. Pigotti attributed it to growth form. The evidence that you adduce for nutrients could be considerably strengthened by a quantitation of the nutrients actually added or by evidence from the water quality data. I lean in favor of nutrients because that explains why the stimulatory effect of sediments was entirely independent of the quantity deposited. Since all flumes were fed from a single tank, the nutrient exposure would have been the same across all flows and therefore across all levels of bed sediment. Finally, it should be pointed out that if nutrients were responsible, this is not a widely applicable result because most sediment inputs do not originate from lake beds.
**R:** **Paragraph rewritten. We have corrected the citations. Following the reviewer advice, we have strengthened the fertilisation effect including in methods section the amount of nutrients contained in sediments pulses and we have pointed the unusually nutrient concentration of our sediments. Lines 297-313.**

Line 292 “but this effect was smaller at low flows” is perhaps technically correct but misses the obvious: that with sediment addition the negative effect of higher velocity disappeared. Framed in this latter way, the next sentence (lines 292-294), which I cannot understand, becomes unnecessary.

**R: This paragraph has been rewritten and the sentence removed. Line 314-320.**

Line 297: So how might the sediments have increased the resistance of algae to higher flow? This question needs answering and I suggest the answer lies in the nutrient effect. Horner and Welch (1981) found that the optimum velocity for algal growth increased with increasing phosphorus concentration. Basically, at higher velocities, the higher nutrients allow the algae to grow faster than they are sheared. In your case, the added nutrients may have shifted the optimum upward enough to eliminate the negative velocity effect.
**R: Paragraph rewritten. We have follow the reviewer suggestion about optimum velocity for algal growth to discuss our results. Line 314-320.**

Line 299 In what sense? I’m confused.

**R: Removed.**

Lines 308-313. The higher metabolic efficiency at low flows in the presence sediment, where there is more chlorophyll, cannot be related to self-shading if we take chlorophyll as an indicator of biomass. Under self-shading, the ratio of GPP to biomass goes down, not up. In the absence of sediment, the increase in metabolic efficiency with flow is associated with a loss of chlorophyll (biomass), so in this case, the trend is consistent with self-shading.

**R: Paragraph rewritten. We have follow the reviewer comment and we have corrected the metabolic efficiency discussion focusing on self-shading for no-sediments treatment and silt-shading for sediments treatment. Line 324-333.**

------------------------------------------------------------------------------------------------------------------------------

Reviewer #2: Review: Perez-Calpe et al. interactive effects of flow and fine sediments on stream biofilms

Overall I enjoyed reading this study, and only have one significant recommendation related to how the authors treat the concept of scouring flows in the context of their replicated stream channels. I think the author’s conclusions are valid and support what has previously been published, but am not convinced that there are significant new insights provided. They certainly make a case that the interaction between suspended and deposited sediments and their subsequent contribution to nutrient loading and periphyton metabolism is important to consider. E.g., not all sediment is created equal. They also provide evidence that the way in which sediment is deposited onto the biofilm is important, but it is a bit challenging to apply these results to the ‘real world’ because of some experimental design artifacts. Finally, I DO believe these concerns can be addressed and that the manuscript would be valuable to readers in this subdiscipline.

Abstract
Line 34: Why would lower water velocity lower sediment deposition? Doesn’t make sense to me. Faster water velocity should entrain more sediment and keep the tiles clearer of sediment deposition?
**R: Sediment deposition results from the balance between inputs, which increase with discharge, and outputs, which increase with flow velocity (i.e., with discharge). The exact outcome will depend on the details of experimental settings. In our case, flow velocity in all channels was rather slow, so the effect of input differences among channels prevailed over the effect of output differences. We have clarified this in the methods and results sections.**

Paragraph ending with line 78: I really liked this summary of the varied and complex interactions that occur with biofilm metabolism as a result of flow modification. Excellent.

Line 99: change ‘was’ to ‘were’.

**R: It is the bottom that was covered, so we left the text as it was. Line 101.**

Line 103: were grazers kept from incubating tiles? Please clarify.

**R: It has been clarified in the text. Line 106.**

Line 110: Did you measure/quantify dissolved nutrients? Seven days of recycled water could lead to significant nutrient reduction although you may have quantified this.

**R: Yes, we measured dissolved nutrients before and after renewing water (line 141). We have added more information about parameters on results section (line 204).**

Line 121: A couple of concerns here: in a ‘natural’ flood, sediment would remain in suspension for a longer period of time and abate during the falling limb of the hydrograph. By not resuspending the sediment additions, I’m not sure how much of an effect you would have, although this may become evident when looking at your AFDM or turbidity data (e.g., suspended sediment was deposited within the stream channels based on water velocity characteristics). Another concern with the sediment addition is related to dissolved and sediment-derived nutrients. As you noted in the introduction, sediment comes hand-in-hand with nutrients that can be released and utilized by the biofilm. So, how do you know that the results you obtained weren’t due to differences in dissolved nutrients between treatments? This is an important, potentially confounding factor in your study design. Perhaps you include this in your analyses.

**R: About the first concern, we agree with the reviewer with sediment deposition on natural floods, but in addition to flood-related pulses, turbidity pulses are quite common in our streams, for instance, when forestry machinery crosses over stream channels. We have clarified this in the text. Moreover, in our experiment we focused on the effects of the amount of sediment deposited and not into the effects of turbidity, for this reason we quantified the amount of sediment per unit of surface.**

**Regarding the second concern, we concluded that the differences between treatments were produced by nutrients derived from sediments, as shown by their leachates. We now describe and discuss this in greater detail.**

Line 191: Interesting! Why do you think conductivity was lower in the sediment treatment? This doesn’t make sense to me unless the sediment was adsorbing dissolved ions.

**R: This is interesting indeed. It is possible that the sediment was adsorbing some dissolved ions. Unfortunately, our data do not allow us to test the potential relevance of this mechanism. Line 199.**

Line 214: Table 2. Is there a way to indicate the direction the significant interactions? For example, deposited sediment increased with increasing flow, etc.

**R: Following the reviewer suggestion, we have added a new column in Table 2, with the sign of the coefficient when the source of the variation is significant.**

Line 236: Although I agree with your interpretation of the data—namely that sediment deposition on the tiles increased metabolism, I don’t think you can claim that higher flows resulted in scouring. If that were the case, sediment deposition on the tiles would be lower in the high velocity treatments. Right? I think you can fix this by changing the wording here, and would strongly advise that you restructure the materials/methods too. I’d refrain from talking about scouring flows, and instead simply note that sediment deposition in channels with higher flow and velocity was an intentional artifact of your sediment/no-sediment treatment. It seems to me that when you dosed your feeder tanks with sediment, there was more sediment deposited on tiles in channels with higher flows because they were pushing more sediment (mass per unit time) over the channel. Does this make sense? Obviously there is no scouring going on at these velocities or you wouldn’t see this pattern.
**R: We have clarified the blocks functioning in the methods section (line 126). Results section has been rewritten (line 223-231), and we replaced “scour” with “slough” in the text.**

Line 244: change ‘showing’ to ‘showed’.

**R: Done. Line 259.**

Line 245: I am looking at Figure 3, and although I appreciate your statistical inference, I’m wondering if you believe it is ecologically significant? E.g., is the pattern indicating an ecological response, or is it an artifact of the experimental design and indicative of significant variability in the data?
**R: Following the comments of the other reviewer, we detected an error in the figure 3 (now Fig 4). We have change the adjustment lines and the interpretation. We hope answer to his comment. Regarding its ecological relevance, we believe it is, although it might not occur when experimental conditions differ very much from ours.**

Line 276 and preceding paragraph: Excellent review of some of the key papers looking at biofilm/velocity relationships.

Line 317: Discussion—Very nice. In particular, I appreciated your thoughtful review of your initial hypotheses. I also appreciated your observation that velocities used in this study were on the low side relative to the literature (aka Biggs et al., etc.).

Minor point: I find that some readers mistake ‘flow’ for ‘velocity’. I’d recommend that you change this to ‘discharge’ throughout.

**R: Following the reviewer suggestion, we have replaced “flow” with “discharge” in all the text.**
