# Supplementary material for: Interactive effects of discharge reduction and fine sediments on stream biofilm metabolism
Source: PLoS One. 2021 Feb 11;16(2):e0246719. doi: 10.1371/journal.pone.0246719 (PMC7877786; doi:10.1371/journal.pone.0246719)
Supplement: Attachment — Submitted filename: Response Reviewers.docx [file pone.0246719.s003.docx]

Dear Dr. McNair,

A new revised version of the manuscript PONE-D-20-22278R1 “Interactive effects of discharge reduction and fine sediments on stream biofilm metabolism” by Ana Victoria Pérez-Calpe, Aitor Larrañaga, Daniel von Schiller and Arturo Elosegi, is attached to your consideration for publishing as an article in PLOS ONE journal.

We are very grateful again to the reviewers for their detailed and positive comments. We have followed closely these comments, as we detail below.

We hope that the present version of the Ms reaches the standards for PLOS ONE journal.

Best regards,

Ana Victoria Pérez-Calpe, PhD student

Faculty of Science and Technology

University of the Basque Country,

P.O. Box 644, 48080 Bilbao, Spain

Tel.: +34 94 601 5939

Email: anavictoria.perez@ehu.eus

Reviewer #1

The paper is greatly improved from the first draft. As a referee I found it highly gratifying to have essentially all of my comments taken seriously and responded to in a substantive way. There do remain a few issues, which are noted below along with minor editorial notes.

I have a new concern, however, which is that the lines in Fig. 2 (formerly Fig. 1) should not have been removed and should be restored. I agree with the removal of the R-squared values but I think it essential to show the solid and dotted lines together with an explanation in the legend that that separate lines imply a significant interaction. The reason is that the mixed-model (LMEM) results provided in Table 2 do not tell the whole story. The significance of the main effect of a covariate says nothing definitive about the significance of the individual within-treatment slopes. A significant interaction says only that the slopes are different.

**R:** **We have restored all the trend lines in Fig 2 and we have added the explanation in the legend.**

In the case of effects on chl-a (where the problem is most serious), the important Points were: (1) in the absence of sediments, discharge had a negative effect, (2) sediments had a positive effect; and (3) in the presence of sediments, the negative effect of discharge vanished. Table 2 tells us only that sediments have a significant main effect and that that there is an interaction with discharge. The text, as a result, appears contradictory, stating first that the no-sediment chl-a declined with discharge (but without a clear statement that this decline was significant), then that the effect of discharge was not significant. It references the interaction but fails to specify that this interaction reflected two slopes, one significantly negative (no-sediments) and the other essentially zero (sediments). Point (3), therefore is lost altogether. Fig.2C, as it stands, is of little help—just a bunch of points that are difficult to interpret. The lines (as on old Fig 1 C) make all the difference. They make the trends easy to see and, more importantly, they provide the reference necessary to report all three Points in the text. In the original submission (with the lines), all three Points were presented in the text. Point (3) must be presented as a Result because it is referenced in the Abstract (line 38) and is addressed in the Discussion (line 316 and following).

**R: We have changed the text to avoid contradictions and to present the third point.**

In the case of deposited sediment (Fig. 2B), the issue is less important, but the text nonetheless suffers from the absence of the within-treatment slopes. If the slopes were available for reference it would be possible to correct the sentence on lines 226-229 to clarify that the discharge affected sediment deposition, but only in the sediment treatment; and to strike the unhelpful last sentence (line 229-231) of the paragraph. (I am afraid that it was my comment to Line 210 of the original submission--“Did deposited sediment really increase with flow in the no-sediment treatment?”—that led to the removal of the trend lines from Fig. 2 and for that I apologize. I didn’t mean to challenge the lines, just the misinterpretation of the Table-2 stats.)

**R: Done**

In the case of metabolic efficiency (Fig. 4) you have retained the slopes (now corrected), which I applaud. Here, they are obviously essential to the presentation. However, the text remains problematic. Although you didn’t see main effects, it seems misleading to say that there was “no significant effect of discharge or sediments” because in the no-sediments treatment there was a clear effect of discharge on efficiency. SAS Proc Mixed run on your dataset shows this effect (the slope of this line) to be significant. I also get that the slightly negative slope for sediments treatment was not significant. For this reason, I would change the phrase on line 216 which reads “in the presence of sediments, increasing discharge resulted in a lower metabolic efficiency” (not significant) to “in the absence of sediments, increasing discharge resulted in a higher metabolic efficiency” (significant).

**R: Changed.**

In the above I have skirted the question of whether the values of the slopes and their significance probabilities should be reported. My sense is that it would be sufficient to report in the text or figure legends simply whether a given slope is or is not significant. I am shaky ground here and am not familiar with lme in R, but I find using SAS Proc Mixed that, where there is a significant interaction, one needs to remove the main effect (discharge in this case) from the model statement to get the within-treatment slopes and significance, which otherwise are reported relative to the respective main-effect values.

**R: We understand the concern of the referee here: significant interaction means crossing lines and, thus, the interpretation is linked to the range of the covariate of the analysis. We have interpreted the results based on the models and the figures. Even with a significant interaction term, if we were observing a significant effect of our treatment AND the figure was showing evidence that values in our discharge range were different comparing one treatment with the other, we have stated that difference.**

Other comments:

Keywords: spell out chlorophyll-a

**R: Done**

36: hyphenate: “no-sediments”

**R: Done**

37-38: If there is room, acknowledge that nutrients may explain the sediment effect.
**R: Done**

107: The schematic of Fig. 1 helps greatly. Thank you.
**R: Thanks**

132: I suggest “Sediments leachate” should be “Sediment leachate.” Consider making this change throughout, wherever “sediments” is used as an adjective.
**R: Done**

139: strike “with”

**R: Done**

139-140. I see a discrepancy here. Are you counting only the ammonium and nitrate to get the 20.9 mg added? If so, please clarify. The phosphorus looks right.

**R: Yes, this value is the result of sum ammonium and nitrate.**

140: I think the quantities of N and P would be better expressed as concentrations, i.e., by dividing by the block water volume if I understand correctly.

**R: As the water is recirculating, for some purposes it may be more interesting to express these quantities as total mass. Therefore, we express them in both ways: mass and concentration**.

140: “to the each” should read “to each”

**R: Done**

206-209. I would not call the changes in SRP, NO3, and DOC “small”. The increase in SRP, for example, was about equal to the average concentration. It may be useful to note that most of the increases appeared to come from the new water, rather than from the sediments.

**R: Clarified.**

261: As noted above, should read: “in the absence of sediments, increasing discharge resulted in a higher metabolic efficiency”.

**R: Done**

263: Fig. 3. Need to express oxygen in units per area.

**R: Changed and S1. Dataset has been corrected too.**

316: “consisted on” should be “consisted of”.

**R: Done**

317: Change “shows…conditions” to “is consistent with a fertilizing effect that is stronger than the sloughing effect” because the fertilizing effect is only suggested, not established, and the response doesn’t show anything about the fertilizer effect if it doesn’t exist.

**R: Changed**

321: Baatrup et al. should be “Baattrup-Pedersen et al.” I hope you are right on the point made in this sentence; I could not understand that paper.

**R: We have corrected the reference. We also have checked the paper and the sentence about it is correct.**

332-333: This still needs work. We might expect sediment shading to reduce metabolic efficiency, but in your case, the metabolic efficiency was NOT reduced; it was the same as no-sediments at high flows and higher than no-sediments at low discharge. My explanation: Sediment had a greater stimulatory effect on GPP than on chl a . At low flows this more than compensated for any sediment-shading effect, so sediment efficiency was greater than no-sediment efficiency. At high flows the stimulatory effect of the sediments was matched by the enhancing effect of the reduced self-shading in the no-sediment treatment, so the efficiencies were about equal.

**R: Modified.**

Reviewer #2: 

I appreciate the author’s due-diligence responding to reviewer suggestions and questions and the manuscript is improved. I particularly appreciate the clarification presented in Table 2, coupled with Figures 3 and 4 and think this makes for a compelling argument for the interactive effects of sediment-derived nutrient addition and biofilm stimulation (e.g., bulk rates of GPP and NCM) vs. photosynthetic efficiency (GPP/chl-a ratio). Figure 1 is also a helpful addition.

Specific comments:
Line 139: Please recast for clarity. Delete ‘with’ and ‘the’ (line 140).

**R: Done**

Line 302-305: Clarify how you quantified ‘type of biofilm’. This is confusing as you state at the beginning of the sentence that you did not study algal composition.

**R: Changed.**
